# 6-Formylindolo[3,2-b]carbazole (FICZ) Enhances The Expression of Tumor Suppressor miRNAs, miR-22, miR-515-5p, and miR-124-3p in MCF-7 Cells 

**DOI:** 10.22074/cellj.2020.6549

**Published:** 2019-09-08

**Authors:** Keivan Mobini, Elham Banakar, Gholamhossein Tamaddon, Afshin Mohammadi-Bardbori

**Affiliations:** 1 Department of Pharmacology and Toxicology, School of Pharmacy, Shiraz University of Medical Sciences, Shiraz, Iran; 2 Diagnostic Laboratory Sciences and Technology Research Center, School of Paramedical Sciences, Shiraz University of Medical Sciences, Shiraz, Iran

**Keywords:** Aryl Hydrocarbon Receptor, Estrogen Receptor Alpha, 6-formylindolo[3,2-b]carbazole, Tumor Suppressor miRNAs

## Abstract

**Objective:**

microRNAs (miRNAs) play bifunctional roles in the initiation and progression of cancer, and recent evidence has confirmed that unusual expression of miRNAs is required for the progress of breast cancer. The regulatory role of aryl hydrocarbon receptor (AhR) and its endogenous ligand, 6-formylindolo[3,2-b]carbazole (FICZ) on the expression of tumor suppressor miRNAs, miR-22, miR-515-5p and miR-124-3p, as well as their association with the estrogen receptor alpha (ERα) were the aims of this study.

**Materials and Methods:**

In this experimental study, the expression levels of *miR-22, miR-515-5p, miR-124-3p* and *miR-382-5p* in MCF-7 cells were determined using the quantificational real time polymerase chain reaction (qRT-PCR) assay.

**Results:**

Our results revealed that *miR-22, miR-515-5p,* and *miR-124-3p* expressions were significantly increased in cells transfected with ERα siRNA. Our data also showed that *miR-22, miR 515-5p,* and *miR-124-3p* expression levels were significantly increased following FICZ treatment. Here, we found that AhR/ERα cross-talk plays a critical role in the expression of *miR-22, miR-515-5p* and *miR-124-3p* in MCF-7 cells.

**Conclusion:**

Overall, our data demonstrated that FICZ, as an AhR agonist could induce the expression of tumor suppressor miRNAs, miR-22, miR-515-5p, and miR-124-3p; thus, FICZ might be regarded as a potential therapeutic agent for breast cancer treatment.

## Introduction

Breast cancer as a malignant neoplasm originated from the breast tissues, is still the most common cause of women death worldwide despite advances made in both diagnosis and treatment (1). 

microRNAs (miRNAs) are single-stranded non- coding RNAs with small size that regulate some of biological processes such as cell proliferation, differentiation, migration and apoptosis. miRNAs play roles in post-transcriptional modification of mRNAs by binding to the 3’-untranslated regions (3’-UTRs) through complementary base pairing (2), resulting in cleavage/degradation of the mRNA and consequently, translational repression (3). miRNAs can function as either oncomiRs or tumor suppressors (4). Owing to their potential ability to regulate numerous protein- encoding genes, miRNAs are regarded as a promising new target in the development of clinical treatments (5). miRNAs were found to be over-expressed in various human diseases including cancers (6). *miR-22* regulates estrogen receptor alpha (ERα) target genes by direct binding to ERα 3’-UTR region (7) through both destabilizing mRNA and inhibiting translation (8). *miR-22* increases the radiosensitivity of breast cancer cells and inhibits tumorgenesis by targeting Sirt 1 (silent information regulator 1) (9). Moreover, miR-22 down-regulates the proto-oncogene ATP citrate lyase which inhibits the growth and metastasis of breast cancer cells (10). 

*miR-515-5p* controls cancer cell migration through modulation of MARK4 (microtubule affinity- regulating kinase 4) 3’-UTR region (11). The *miR-124* expression is significantly suppressed in breast cancer cells (12). *miR-124-3p* appears to be a tumor suppressor in breast cancer cells and it acts via targeting CBL (Cbl proto-oncogene, E3 ubiquitin protein ligase) (13). However, the molecular pathways underlying *miR-124* modulatory actions in breast cancer cells are not fully understood. Cyclin-dependent kinase 4 (CDK4), a master regulator of the cell cycle belonging to the CDK family (14), is identified as a major oncogenic driver among the cell cycle components (15); also, CDK4 has been found in several tumor types including breast (16) and lung cancers (17). It was shown that CDK4 is a target of miR-124 (12). 

Development of breast cancer is closely associated with estrogen levels in the body. UDPglucuronosyltransferase (UGT) is an important class of phase 2 drug metabolizing enzymes that plays a pivotal role in detoxification of steroid compounds. UGTs eliminate estrogen hormones and influence estrogen signaling pathway (18). UGT2B isoforms are involved in regulating cell proliferation in human cancer cells. The UGT2B4, 2B7 and 2B15 isoforms are also involved in the glucuronidation of biologically active lipids (19). *miR-382-5p* regulates UGT2B15 and UGT2B17 isoforms (20). The Ras GTPase superfamily member RERG (Ras-related and estrogen- related growth inhibitor) reduces breast cancer cells proliferation and tumor formation. RERG was shown to play a regulatory role in the Ras/ERK pathway. *miR-382-5p* directly represses RERG; therefore, *miR-382- 5p* promotes viability, survival, migration and invasion of breast cancer cells (21).

The aryl hydrocarbon receptor (AhR) belongs to the family of basic helix-loop-helix nuclear transcription factors (22). The AhR downstream targets, cytochrome P450 (CYP1) isoforms, play bifunctional roles in detoxification or bioactivation of carcinogens, xenobiotics, and physiological compounds such as benzo(a)pyrene and estradiol (23). At the cellular level, AhR has functional interactions with signaling pathways governing cell proliferation and cell cycle, cell morphology, cell adhesion and cell migration (24). 6-formylindolo[3,2-b]carbazole (FICZ), a derivative of tryptophan (Trp) amino acid, is an ideal substrate for CYP1A1, 1A2, and 1B1 (25). FICZ also binds the AhR with the highest affinity known to date and thus, it reveals the characteristics of an endogenous signaling molecule (26-28). FICZ stimulates AhR-mediated activation of drug metabolizing enzymes such as CYP1A1 that end up its activity by generating a negative feedback control of its action (22, 25, 27-29). 

This study was designed to reveal effects of FICZ, as an endogenous AhR ligand, on the expression levels of *miR22, miR-515-5p, miR-124-3p* and *miR-382-5p *in MCF-7 breast cancer cell line.

## Materials and Methods

### Chemicals

6-formylindolo[3,2-b]carbazole (FICZ) was purchased from Syntastic AB, Sweden. 1-methyl-N[ 2-methyl-4-[2-(2-methylphenyl)diazenyl] phenyl]1H- pyrazole-5-carboxamide (CH223191) and 17ß-Estradiol (E2), dimethyl sulfoxide (DMSO) were bought from Sigma-Aldrich, Germany. All cell culture reagents and media were purchased from Invitrogen. 

### Cell culture and chemical treatments

In this experimental study, MCF-7 cells were maintained in 10% fetal bovine serum (FBS)supplemented Dulbecco’s modified Eagle’s medium (DMEM) containing 100 µg/mL streptomycin, and 100 IU/mL penicillin under an atmosphere containing 5% CO_2_ at 37°C. Cells were treated with desired concentrations of chemicals, after replacing the growth medium with fresh medium without FBS. The final concentration of DMSO was 0.1% (v/v). 

### Small interfering RNA treatments

SiRNA against ERα (Santa Cruz Biotechnology, CA, USA) was used for the targeted knockdown of ERα protein expression. Non-targeting scrambled siRNA (Santa Cruz Biotechnology, USA) was used as a control. MCF-7 cells were seeded in 6-well plates and grown in an antibiotic-free medium containing 5% FBS. At 50-60% confluence, the cells were transfected with 100 nM ERα siRNA or scrambled siRNA using lipofectamine 2000 (Invitrogen, USA) in 1 ml of transfection medium (Santa Cruz Biotechnology, USA). After 5 hours, the medium was replaced with fresh medium and 3 hours later, the cells were treated with DMSO, FICZ (1 nM), E2 (10 nM), and CH223191 (10 nM) for 18 hours. 

### RNA extraction and cDNA synthesis of miRNAs 

The TRizol reagent (Invitrogen, Carlsbad, CA, USA) was used for isolation of total RNA according to the manufacturer’s instructions; then, total RNAwas reversely transcribed into cDNA by using the RT microRNA Kit (EXIQON, Denmark). The ERα mRNA in the cells was quantified by using the following primers: 

F: 5´-GTTCTTAGTGGCACATCTTCTG-3´

R: 5´-GAATCCTCACGCTTAGTAACATAG-3´. 

Real-time reverse transcription polymerase chain reaction (RT-PCR) amplification consisted of 40 cycles (95°C for 5 seconds, 63°C for 20 seconds, and 72°C for 30 seconds) after an initial denaturation done at 95°C for 5 minutes in an ABI StepOne™ real-time quantitative PCR system. The fold change of the miRNA expression was calculated by using the 2^-ΔΔCt^ method after normalization against the 5S rRNA (used as internal control) expression. 

### Statistical analysis

Statistical significance was determined by one-way ANOVAand Tukey test. The results are expressed as means ± SD for at least three separate (replicate) experiments for each treatment group in the *in vitro* studies. P<0.05 were considered statistically significant.

## Results

### Effect of ERα on the expression of *miR-22, mir-515-5p, miR-124-3p* and *miR-382-5p*, in MCF-7 cells

**Fig.1:**
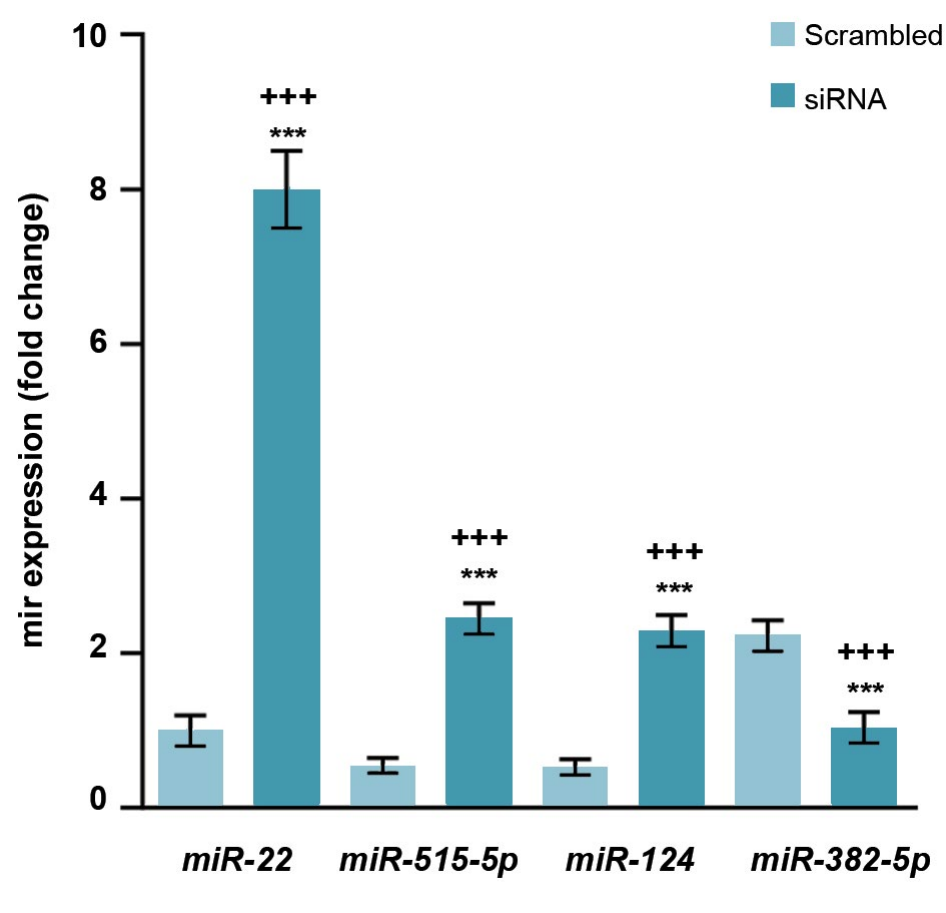
Expressions of *miR-22, miR-515-5p,* and *miR-124-3p* were inversely correlated with ERα in MCF-7. The cells were treated with E2 (10 nM) and the levels of *miR-22, miR-515-5p, miR-124-3p* and *miR-382-5P* were measured using real-time RT-PCR. Values are expressed as means ± SE. Asterisks denote significant differences (***; P<0.001) between control and other treated groups and significant differences (+++; P<0.001) between cells treated with siRNA and those treated with scrambled siRNA. ERα; Estrogen receptor alpha and RT-PCR; Real-time reverse transcription polymerase chain reaction.

In this study, MCF-7 cells were treated with E2 (10 nM) and our results revealed that *miR-22, miR-515-5p,* and *miR-124-3p* expressions were significantly increased and miR-382-5p were decreased. The expression of *miR-22, miR-515-5p,* and *miR-124-3p* were respectively 8, 2.46, and 2.29 times higher in the ERα-silenced cells than scrambled ones (Fig .1). 

### Effect of AhR on the expression of *miR-22, mir-515-5p, miR-124-3p* and *miR-382-5p,* in MCF-7 cells

MCF-7 cells were treated with an AhR agonist, FICZ (1 nM) or an AhR antagonist, CH223191 (10 nM) either alone or in combinations, our data showed that the *miR-22, miR515-5p,* and *miR-124-3p* expression levels were significantly increased by FICZ and CH223191 treatments. The expression of *miR-22, miR515-5p,* and *miR-124-3p* in FICZ, CH223191 and FICZ+CH223191 treated groups were respectively 12.55, 7.94, 7.46; 4.75, 2.21, 3.7 and 8.69, 2.29, 5.27 times higher than the control group (Figes.2-4). *miR-382-5p* expression levels significantly decreased in cells treated with FICZ+ CH223191 (Fig .5). 

**Fig.2:**
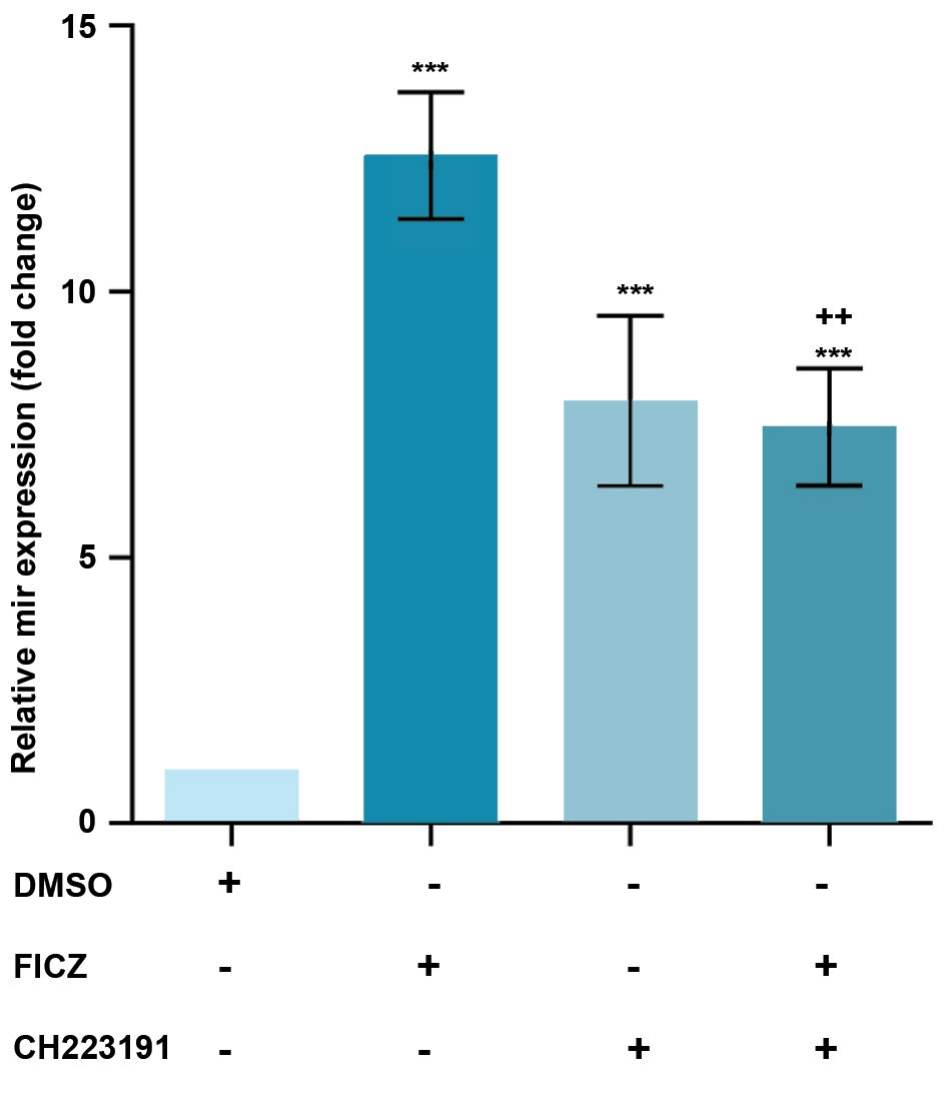
Expression of *miR-22* was AhR-dependent in MCF-7. The quantitative RT-PCR analysis showed that the expression level of *miR-22* was much higher in the cells treated with FICZ in comparison to control. Values are expressed as mean ± SE. Asterisks denote significant differences (***; P<0.001) between control and other treated groups and significant differences (++; P<0.001) between cells treated with FICZ and the cells treated with FICZ+ CH223191. AhR; Aryl hydrocarbon receptor, RT-PCR; Real-time reverse transcription polymerase chain reaction, and FICZ; 6-formylindolo[3,2-b]carbazole.

**Fig.3:**
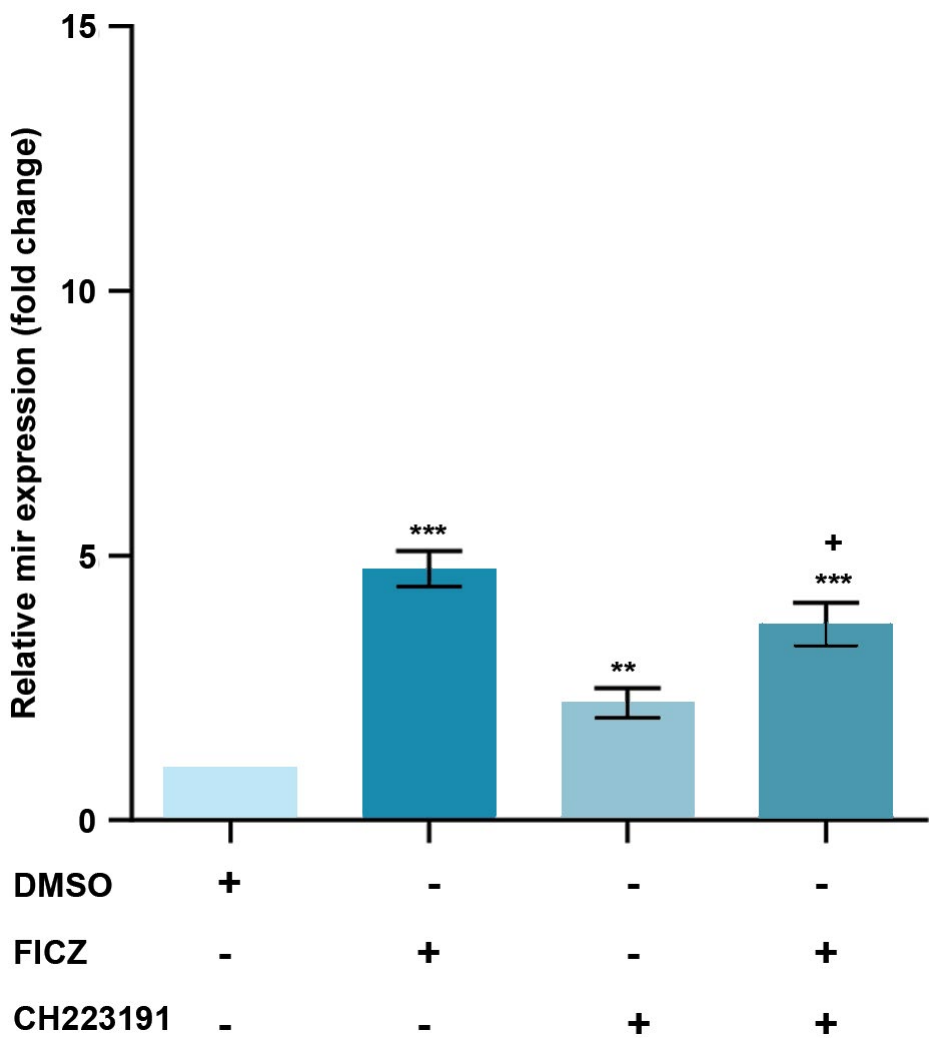
Expression of *miR-515-5p* was AhR-dependent in MCF-7. The quantitative RT-PCR analysis showed that the expression levels of *miR-515- 5p* were much higher in the cells treated with FICZ in comparison to control. Values are expressed as mean ± SE. Asterisks denote significant differences(**; P<0.01 and ***; P<0.001) between control and other treated groupsand significant differences (+; P<0.05) between cells treated with FICZ andthe cells treated with FICZ+ CH223191. AhR; Aryl hydrocarbon receptor, RT- PCR; Real-time reverse transcription polymerase chain reaction, and FICZ; 6-formylindolo[3,2-b]carbazole.

**Fig.4:**
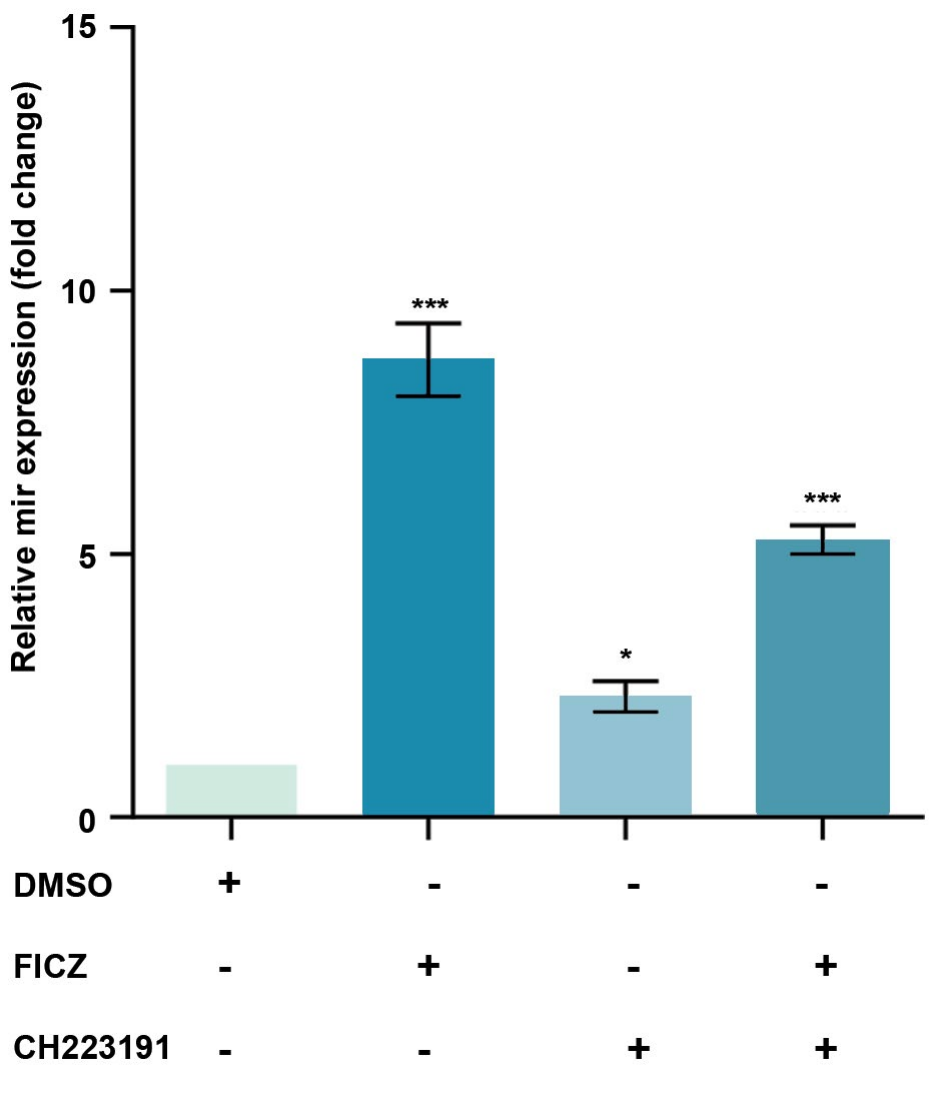
Expression of *miR-124-3p* was AhR-dependent in MCF-7. The quantitative RT-PCR analysis showed that the expression levels of *miR- 124-3p *were much higher in the cells treated with FICZ in comparison to control. Values are expressed as mean ± SE. Asterisks denote significant differences (*; P<0.05 and ***; P<0.001) between control and other treated groups. AhR; Aryl hydrocarbon receptor, RT-PCR; Real-time reverse transcription polymerase chain reaction, and FICZ; 6-formylindolo[3,2-b] carbazole.

**Fig.5:**
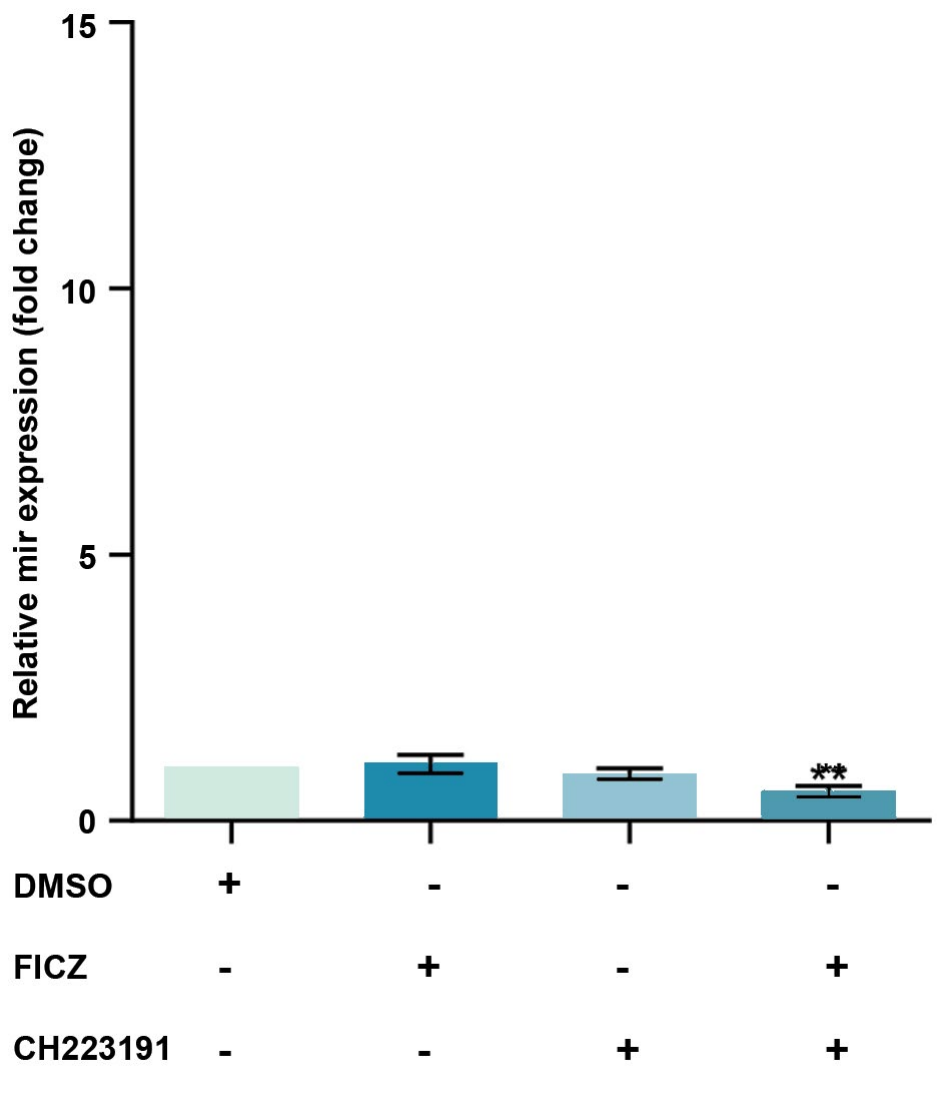
Expression of miR-382-5p was AhR-dependent in MCF-7. The quantitative RT-PCR analysis showed that the expression levels of miR- 382-5p were much lower in the cells treated with FICZ+CH223191 in comparison to control.. Values are expressed as mean ± SE. Asterisks denote significant differences (**; P<0.01) between control and other treated groups. AhR; Aryl hydrocarbon receptor, RT-PCR; Real-time reverse transcription polymerase chain reaction, and FICZ; 6-formylindolo[3,2-b] carbazole.

## Discussion

miRNAs are stable biomarkers as they have high stability in extreme conditions such as low pH and high temperatures (30) and are used as prognostic and therapeutic tools for breast cancer (31). 

In ERα silencing cells, we observed significantly increased expression levels of *miR-22, miR-515-5p,* and *miR-124-3p*. Furthermore, FICZ treatments led to over- expression of *miR-22, miR-515-5p,* and *miR-124-3p*. 

*miR-22* regulates ERα target genes by direct binding to the ERα 3’-UTR region (7) through both destabilizing and inhibiting translation of mRNA (8). *miR-22* represses CD147 expression by directly targeting the *CD147* 3’UTR site. miR-22 also indirectly participates in the CD147 modulation by down-regulating Sp1. Indeed, *CD147 *is overexpressed in breast cancer tissues, and its high expression is correlated with tumor invasion and metastasis (32). The transcription factors Sp1 could bind to the *CD147* promoter and enhance its expression as well. In addition, low miR22 levels are significantly associated with poor differentiation of breast cancer cells. Furthermore, *SIRT1* (Sirtuin1) expression levels are significantly up-regulated in breast cancer tissues. Since *miR22* has suppressive effects on breast cancer cells via targeting *SIRT1*, miR22/SIRT1 axis may be used as a novel and potential therapeutic target for breast cancer treatment (33).

Sphingo kinase-1 (SK1) mediates cell proliferation in cancer cells. *miR-515-5p* targets SK1 and inhibits breast cancer cells growth. Previous studies reported that SK1 mediates estrogen-dependent tumorigenesis in MCF-7 cells and estradiol down-regulates *miR-515-5p* expression but increases SK1 activity (34). *miR-124* targets Slug (SNAI2, transcriptional repressor of E-cadherin) and regulates epithelial-mesenchymal transition and metastasis of breast cancer cells (35). miR-124 also suppresses breast cancer cells growth and motility by targeting CD151 (36). Moreover, *miR-124-3p* inhibits tumor metastasis by inhibiting *PDCD6* expression. In this regard, miR-124-3p/PDCD6 signaling axis may be a potential target for treatment of patients with advanced breast cancer. 

Our results showed that ERα silencing significantly led to *miR-382-5p* down-regulation. *miR-382-5p* targets UDP-glucuronosyl transferases (UGTs) (20) which are involved in the detoxification of estrogen derivatives (18). Thus, *miR-382-5p* down-regulation may enhance estrogen detoxification. One of the new findings of the present study was that ERα silencing or FICZ treatment led to up- regulation of *miR-22, miR-515-5p,* and *miR-124-3p*. ERα suppresses Drosha (one of the main processing enzymes in miRNA biogenesis) activity in MCF-7 cells (37). Therefore, we suggest that silencing ERα may enhance tumor suppressor miRNAs such as *miR-22, miR-515-5p* and *miR-124-3p*. 

A number of studies reported that AhR-ARNT complex may reduce ERα-mediated transactivation (38) either directly by binding the inhibitory site of XRE (iXRE) or by employing shared coactivators (39). 

Some reports also indicated that ERα can be activated by AhR agonists, but not by AhR antagonists (40). However, AhR antagonists may exhibit a partial effect.

## Conclusion

Our data demonstrated that the overexpression of tumor suppressor miRNAs including *miR-22, miR-515-5p,* and *miR-124-3p* by FICZ, as an AhR agonist, might be considered a potential therapeutic approach against breast cancer. 
